# Dynamics of Actin Waves on Patterned Substrates: A Quantitative Analysis of Circular Dorsal Ruffles

**DOI:** 10.1371/journal.pone.0115857

**Published:** 2015-01-09

**Authors:** Erik Bernitt, Cheng Gee Koh, Nir Gov, Hans-Günther Döbereiner

**Affiliations:** 1 Institut für Biophysik, Universität Bremen, Bremen, Germany; 2 School of Biological Sciences, Nanyang Technological University, Singapore, Singapore; 3 Department of Chemical Physics, Weizmann Institute of Science, Rehovot, Israel; University of British Columbia, CANADA

## Abstract

Circular Dorsal Ruffles (CDRs) have been known for decades, but the mechanism that organizes these actin waves remains unclear. In this article we systematically analyze the dynamics of CDRs on fibroblasts with respect to characteristics of current models of actin waves. We studied CDRs on heterogeneously shaped cells and on cells that we forced into disk-like morphology. We show that CDRs exhibit phenomena such as periodic cycles of formation, spiral patterns, and mutual wave annihilations that are in accord with an active medium description of CDRs. On cells of controlled morphologies, CDRs exhibit extremely regular patterns of repeated wave formation and propagation, whereas on random-shaped cells the dynamics seem to be dominated by the limited availability of a reactive species. We show that theoretical models of reaction-diffusion type incorporating conserved species capture partially the behavior we observe in our data.

## Introduction

The polymerization of the structural protein actin from its monomeric to its filamentous state accounts for fundamental aspects of the dynamic cell shape [1]. Changes of the cell morphology can therefore be mainly understood by deciphering the underlying actin machinery. In the organization of actin dynamics, waves of protein activity play a central role and have become a field of intense research recently [2]. The prominence of waves for cell morphodynamics and motility seems to be a conserved property among different cell lines and organisms. Popular model systems include *Dictyostelium discoideum*, human neutrophils, and rat tumor mast cells to name but a few [3–5]. The subject of this work is a type of actin wave termed Circular Dorsal Ruffles (CDRs). These are actin-based structures of vertical extension that form and propagate as soliton-like waves at the dorsal side of a large number of cell types such as fibroblasts, epithelial cells, macrophages, glial cells, and aortic smooth muscle cells [6–10]. CDRs were named as such based on their often ring-like appearance and the fact that the membrane at CDRs has ruffle-like morphologies, as visible in scanning electron micrographs, much like peripheral ruffles at the cell edge [11, 12]. Their biological function is still debated, but CDRs clearly serve endocytotic purposes [7]. The fact that several pathogens are known to hijack CDRs as a mechanism of gate opening through the cell membrane brought CDRs to the center of interest of the medical community [12]. CDRs have been comprehensively characterized in terms of protein composition [7, 8], but the mechanism that orchestrates the protein interplay in CDRs remains puzzling.

In this work, we present a detailed study on CDR phenomenology and quantitative analysis of wavefront dynamics that allows us to characterize several properties of the mechanism underlying CDRs. In this respect, we visually identified and quantitatively confirmed features characteristic of current theoretical models of actin waves. Comprehensive theories for the description of actin gels from fundamental properties have been formulated [13–15]. However, most models for description of actin waves focus on a limited number of molecular key-players. A recent review by Allard and Mogilner classifies theories underlying actin wave description into models in which actin is an autocatalytic species that promotes its own growth and into models in which actin only responds to fields of regulatory proteins [2]. The latter are further sub-divided into models of classical reaction diffusion type and into models that also contain morpho- or mechanosensitive species. As an example of a reaction diffusion system, the interactions between the Scar/WAVE complex and actin have been shown to organize into actin waves. In these, Scar/WAVE triggers actin polymerization, but is in turn inactivated by polymerized actin [4]. Actin autocatalysis, supported by the Arp2/3 complex, and the treadmilling properties of actin filaments, on the other side, provide an intrinsic propagator of actin waves as has been shown in *D. discoideum* [3]. In these cells, no Scar/WAVE was required for propagating actin waves. In the organization of actin waves into patterns that allow cells to function, coupling of the actin wave machinery and oscillations of species such as calcium seems to play important roles [5].

For the description of CDRs, there are currently two models, one based on a reaction-diffusion system and the other based on morphosensitive actin factors. The model by Zeng et al relies on an antagonistic reaction-diffusion scheme between the rhoGTPases Rho and Rac [16]. The activity states of these two proteins thereby compete for the organization of actin into either stress fibers or into a meshwork as found in lamellipodia and CDRs. In contrast, Peleg et al consider a system that incorporates membrane-bound proteins that are curvature-sensitive effectors of actin [17]. The curvature of the membrane thus plays a crucial role in this model.

Even though these two studies show that both models support the formation and propagation of actin waves, a comparison to the detailed dynamics of CDRs obtained by live cell imaging is missing. With this work, we show that CDRs on fibroblast cells cover a much wider range of behavior than currently described in the literature on CDRs; among them states of periodic expansion and contraction (“breathing” modes), spiral waves, stalled wavefronts close to cell edges, and mutual wave annihilation upon collision of wavefronts. We analyze the dynamics of CDRs on cells of natural morphology and on cells that we forced into disk-like morphology with centered nuclei. On the latter we find CDRs to propagate laterally between cell nucleus and cell edge while keeping constant sizes. Comparing the data of cells of uncontrolled morphologies and cells of disk-like morphology reveals that CDR size influences their dynamics and that CDRs exhibit very uniform patterns of formation and propagation when their size is fixed. When reviewed in the light of current models of actin waves, CDR dynamics can be understood—without knowledge of molecular details—as waves in an active medium where a limited resource or some other constraint has a large influence.

## Results

### CDRs exhibit behaviors well known from reaction diffusions systems with a strong dependency on local cell morphology

**Figure 1:**
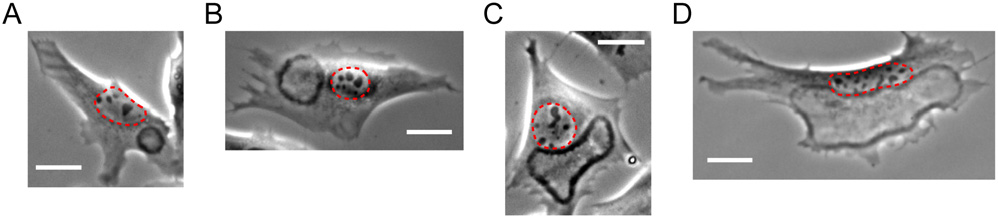
Effects of cell size and morphology on CDR morphology and dynamics. CDRs usually avoid the nucleus region (encircled in red). Cell edge and nucleus therefore define a bounded region available for CDR propagation, which limits the maximal size CDRs can attain. In the panels *A-D* the size of this region is increasing from left to right. The isotropy of CDRs decreases with increasing CDR size, while the tendency to mimic cell morphology increases with CDR size. CDRs in small regions (*A*) cannot extend much, typically forming oscillatory reappearing objects of high isotropy. All scale bars correspond to 25 µm. See S1–S4 Movies (corresponding to panel *A-D* respectively) for the respective characteristic dynamics.

We were interested whether CDRs exhibit, besides formation of expanding rings, other phenomena known to occur in reaction-diffusion systems. Therefore, we performed long-term experiments using a cell line (NIH 3T3 X2) that spontaneously forms CDRs when cultured in cell medium containing Fetal Bovine Serum (FBS). We confirmed that the CDRs formed under these conditions are identical in appearance to CDRs formed in response to Platelet-Derived Growth Factor (PDGF) stimulation (compare Fig. 1, S1–S4 Movies, and S11 Movie with S1 Fig.). The use of PDGF for CDR stimulation is the standard method for studies on CDRs [6–8]. In contrast, using NIH 3T3 X2 cells allowed us to keep the biochemical conditions of the medium fixed in our experiments, refraining from the disturbance of the biochemical surroundings by addition of PDGF. We further confirmed that minima of phase contrast micrographs of CDRs corresponded to maxima in actin concentration (S2 Fig.). The live-imaging data of cells exhibiting CDRs were then scanned systematically for phenomena characteristic for active media.

We found CDRs to grow outward as closed soliton-like structures originating from points. The width of CDRs was typically 5 µm whereas the shape, size, and lifetime strongly depended on cell morphology (compare Fig. 1A–D and, correspondingly S1–S4 Movies). We generally found CDRs to avoid the region of the cell nucleus; we rarely saw CDRs crossing it. CDRs also, trivially, could not propagate beyond the cell edge. CDRs at maximal extension therefore tended to take shapes that reflected the cell morphology (Fig. 1C&D and corresponding S3 and S4 Movies). Since fibroblasts are random-shaped, we consequently found CDRs to largely vary in form, typically strongly differing from circular geometry. The area available for growth and propagation of a CDR, bounded by the nucleus and the cell edge, crucially influenced its dynamics. In the following we describe the phenomena we observed together with the typical size of the region where CDRs emerged.

**Figure 2:**
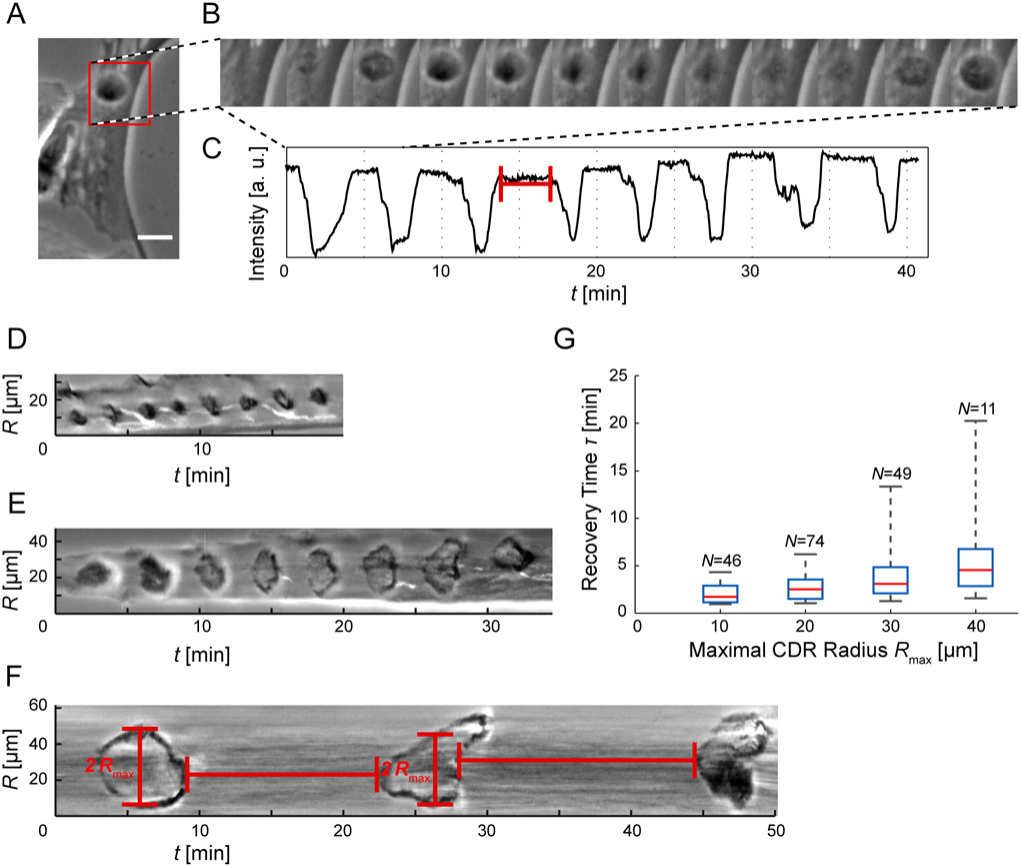
Oscillatory reappearing CDRs. (*A*) CDRs under spatial confinement exhibit oscillatory patterns of pulsating re-appearance (scale bar: 25 µm, full sequence: S5 Movie). (*B*) Stills from the region of interest highlighted red in the time-lapse sequence *A* (Δ*t* = 36 s). (*C*) A plot of the minimal intensity value of the ROI in *A* as a function of time shows CDR events as negative peaks and CDR-free periods, corresponding to the recovery time τ, as plateaus of high intensity. The ROI was smoothed with a Gaussian with *σ* = 2 µm prior to intensity sampling. (*D-F*) Kymographs of CDRs taken along lines crossing CDR origins (see Fig. 4*A* for illustration) show both the recovery time τ between successive events and their radial extension *R*_max_ (cells not shown). (*G*) The recovery times increase with CDR size. The data was binned in *R*_max_-direction (box width: 10 µm) and plotted as boxes with whiskers (red lines: median, upper box edge: 75th percentile, lower box edge: 25th percentile). *N* values denote the number of observations. Note that oscillatory behavior was rare for large CDRs.

In small regions of order twice the characteristic CDR diameter, where there is no space for CDR propagation, we found CDRs to re-appear in a periodical manner at the same position (Fig. 2A&B, S5 and S1 Movies). For these CDRs, the phase of expansion was very short, immediately followed by CDR closure. In sequences of several CDR reappearances, we observed a well-defined frequency of firing (Fig. 2 B&C) that did, however, vary among different cells. The recovery times between two CDRs could be as short as one minute. We also observed repeated formations of larger CDRs on regions of greater spatial extent. However, in such large regions the number of CDRs formed was usually smaller and the frequency lower than for CDRs on small regions (Fig. 2D–G). In no case did we observe concentric wave trains; a succeeding CDR would only form if the preceding CDR either closed or decayed.

**Figure 3:**
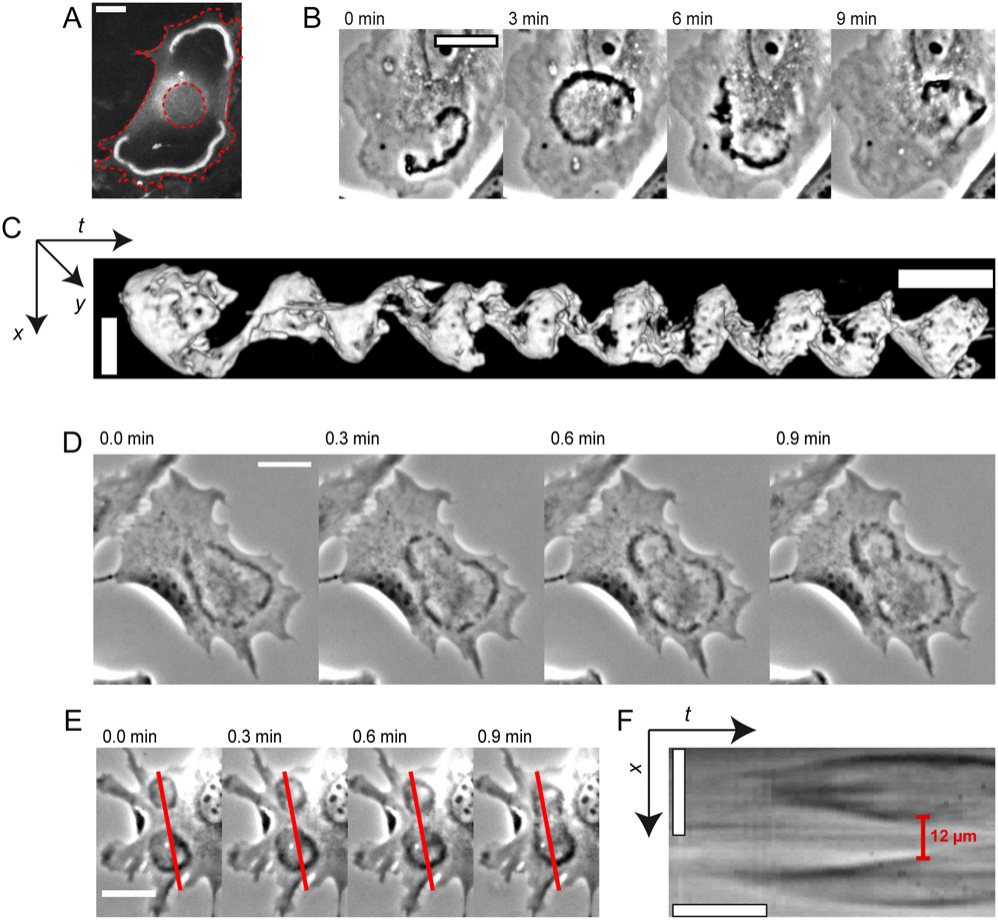
Overview of CDR phenomena. (*A*) CDRs can become trapped close to cell edges; actin staining with pLifeAct–TagGFP2 reveals regions of actin depletion behind wavefronts (S6 Movie). Red dashed lines highlight cell edge and nucleus. (*B*) A CDR propagating as a spiral wave (S7 Movie). (*C*) Iso-surface visualization of the CDR in *B* as an *x-y-t*-projection (see S3 Fig. and S8 Movie). The CDR performed eight full rotations in approximately 70 minutes. (*D*) A CDR dividing into two arc-shaped wavefronts (S9 Movie). (*E* and *F*) Time-lapse sequence of two colliding CDRs and the corresponding kymograph respectively (S10 Movie). The red lines in *E* mark the position were kymographs where sampled. The CDR wavefronts mutually annihilate each other at a distance of approximately 12 µm before they actually make contact. All spatial scale bars correspond to 25 µm. Temporal scale bar in *C:* 10 minutes, temporal scale bar in *F* 1: minute.

In areas larger than twice the characteristic CDR width, CDRs would typically initially grow as circular structures. Upon approach of either the cell nucleus or the cell edge, CDR shapes typically gained asymmetry. With increasing size and asymmetry, we observed a slow-down in the propagation velocity. The subsequent dynamics depended on the overall cell morphology and the position where CDRs emerged on the cell. CDRs would either reverse propagation direction at the nucleus and cell edges and close back to points, decay at maximal extension or they would separate into typically arc-shaped parts that continued to propagate (Fig. 3D, S9 Movie). CDRs that covered the larger part of the cell surface formed closed structures of high anisotropy with life times of up to several ten minutes. Close to the cell edge we observed a persistent stalling of CDR net movement in some cases (Fig. 1D and S4 Movie). This was accompanied by spatial oscillatory fluctuations of CDRs around one fixed position for typically several minutes. We employed fluorescence microscopy to visualize actin dynamics of stalled CDRs and found them to be actin-dense structures surrounded by areas of complete actin depletion (Fig. 3A and S6 Movie).

In cases where more than one CDR formed on the same cell simultaneously, we observed fusion of CDRs when the wavefronts of two CDRs happened to collide. The parts of the colliding wavefronts that moved towards each other thereby annihilated at a typical distance of 12 µm from each other. The remaining parts of the CDRs merged and formed one large CDR (Fig. 3E&F, S10 Movie).

In rare cases, CDRs could be observed to propagate as spirals (Fig. 3B&C, S7 and S8 Movies). The periods for one full rotation varied among different cells between 5 and 10 minutes.

### CDR wavefronts initiate from points and grow with dynamics governed by their current size

**Figure 4:**
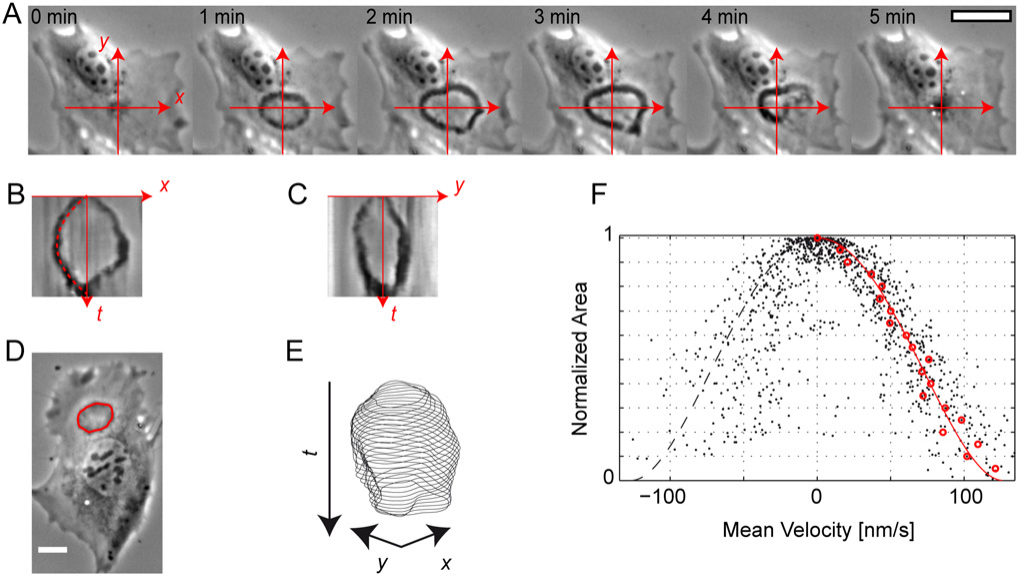
Wavefront dynamics of opening and closing CDRs. (*A-C*) A typical life course of a CDR exhibiting opening and closing (S11 Movie). The coordinate system in *A* is the basis for calculation of the kymographs shown in *B* and *C*. Together with time-lapse sequence *A* these kymographs show the dependency of CDR dynamics on cell features. CDR propagation without encounter of obstacles and absence of instability has a parabolic evolution of the CDR radius with time (red dashed line in *B*: empirical parabola fit). In positive *x*-direction, however, the wavefront becomes unstable and partially decays, leading to an asymmetric profile in kymograph *B*. (*D* and *E*) Using active contours, the wavefronts of CDRs can be tracked yielding sets of contours for each CDR (S12 Movie). (*F*) The contour mean velocity data of 13 CDRs as a function of the normalized area roughly follow one trajectory. Positive velocities correspond to CDR growth and negative velocities to CDR shrinking. Original data points are shown in black, red circles correspond to average velocities calculated using a box median of width 0.05 in normalized area. The red line is an empirical fit function used for extrapolation to a CDR area of zero. See the main text and the *SI Methods* in S1 Text for details. All spatial scale bars correspond to 25 µm.

Knowledge of the typical propagation velocities of wavefronts and their dynamics allows for conclusions on the underlying mechanisms [2]. Further, waves in excitable media often enable the formulation of an eikonal equation that links their curvature to their propagation velocity [18]. Moreover, in models in which curvature-sensitive membrane proteins regulate actin dynamics the local curvature of wavefronts is governing the propagation dynamics. In the following, we analyze the wavefront dynamics of CDRs with respect to their local curvature. Our data set consists of CDRs that followed the pattern of opening (centrifugal CDR growth), reversing and closure (centripetal CDR shrinkage), i.e., where no events like separation or fusion could be observed. See Fig. 4A and the corresponding S11 Movie for an example of a typical member of our data set.

CDRs originated from points and grew as ring-shaped waves. Upon approach of either the cell nucleus or the cell edge, CDRs typically lost isotropy and mimicked the cell shape to some degree (Fig. 4A, S11 Movie). A kymograph analysis revealed that the growth velocity decreased right from the point of CDR formation until CDRs reversed and closed back to points. When propagating unperturbed, without contact with the cell edge or nucleus, CDRs exhibited radius evolutions that were symmetric with respect to the time point of reversal. In a first approximation, this could be described with a parabolic time dependence of the radius (Fig. 4B). Wavefronts with a pronounced halo in phase contrast images, however, tended to decay at maximal extension. See Fig. 4A&B and their captions for a comparison of stable and unstable CDR reversal. Based on the principles of image formation in phase contrast, we assume that halo formation corresponded to a large vertical extension of decaying CDRs. This implies that CDRs tend to decay when the membrane forms high vertical height amplitude.

An encounter of wavefronts with either the cell edge or the nucleus led to disturbed dynamics. Wavefronts either reversed directly or reversed after some time of stalling (Fig. 4C).

Obviously, CDRs can follow different dynamics after propagation reversal. We were curious whether the opening of CDRs still follows a general pattern. Since more often than not, the point of CDR origin and closure were not identical, i.e., the CDR as a whole translocated, a kymograph description was generally not sufficient. Further, frequent deviations from circular shapes of CDRs made velocity measurements using kymographs inaccurate. We thus used the image processing technique of active contours to track CDR wavefronts. We focused on CDRs that had closed shapes of limited complexity, i.e., CDRs similar to those shown in Fig. 1B and Fig. 4A. The sets of contours we obtained this way corresponded to the positions of the CDR wavefronts as a function of time (Fig. 4D&E, S12 Movie). We calculated the local normal velocity of CDR contours and their local curvature. A cross-correlation analysis yielded no correlation between local curvature and local velocity (see S1 Text, *SI Results* and Fig. I therein).

We thus asked whether an integral measure such as the area covered by a CDR could govern its dynamics. A possible mechanism to link area and velocity could incorporate, e.g., a limited resource such as an involved protein or the available membrane area. Since the size of both cells and CDRs varied, we calculated the CDR area and normalized it to its maximal area (S1 Text, *SI Materials and Methods*). We then plotted the mean contour velocity as a function of this normalized area (Fig. 4F). The data points corresponding to the opening of CDRs collapse on a polynomial of fourth order as expected from a parabolic evolution of the mean radius in time (see kymographs in Fig. 4B&C and S1 Text, *SI Materials and Methods*). We used this fit function for extrapolation and found a velocity of 0.13 µm/s at small CDR areas, i.e., directly after CDR formation and shortly before its closure. The fact that we find highest wave velocities directly after wave formation might seem to suggest highest velocity for largest curvature.

### CDRs propagate in a highly regular manner on cells of controlled morphology

**Figure 5:**
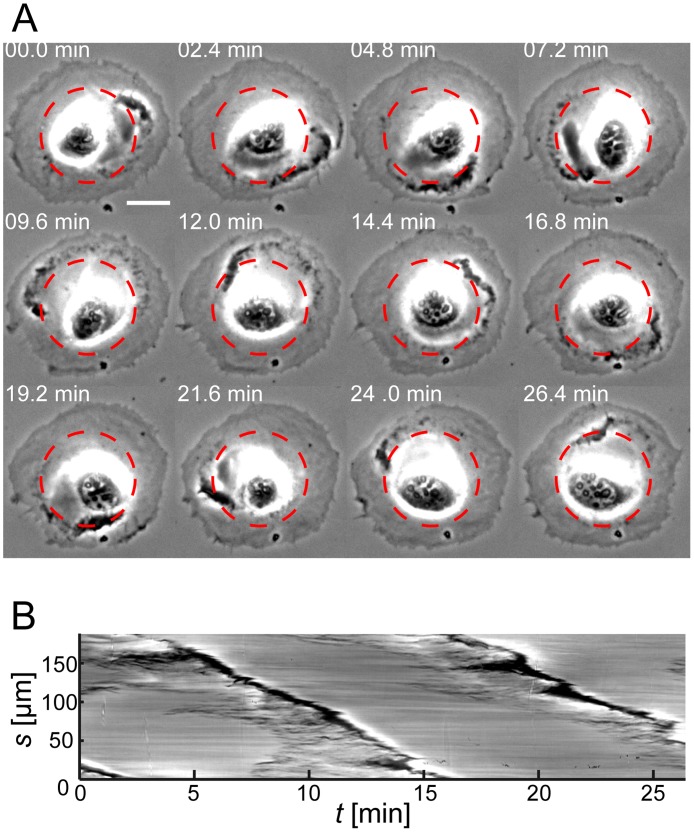
CDR orbiting the nucleus of a cell of disk-morphology. (*A*) Time-lapse sequence of a cell that had disk-like morphology without being plated on a micro protein patch. A CDR propagates between cell edge and cell nucleus, circling the nucleus almost twice (S13 Movie). (*B*) Sampling of the image intensity along the arc length of a circle (highlighted red in *A*) and as a function of time gives rise to a circular kymograph. The nearly constant slope in this kymograph indicates a constant lateral velocity (*v* = 0.21 µm/s) of the CDR. Scale bar in *A*: 25 µm.

**Figure 6:**
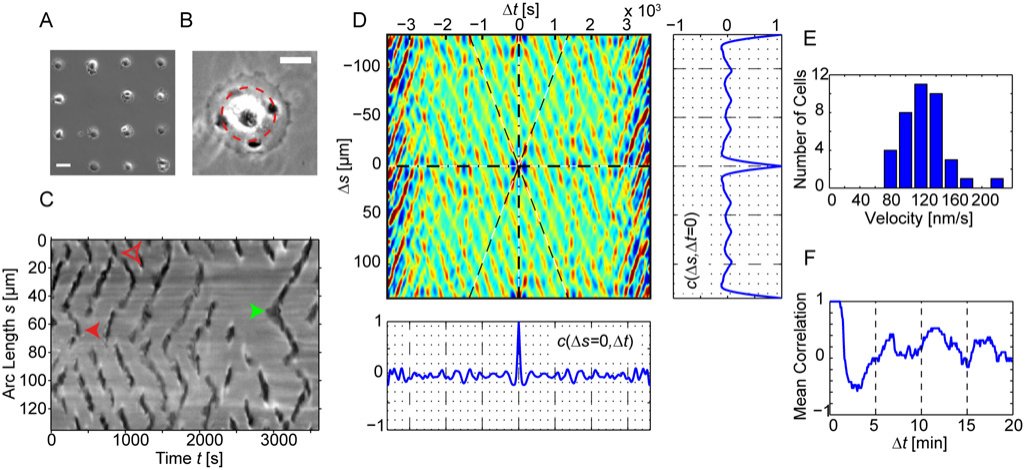
Space-time correlations of CDRs on circular paths. (*A* and *B*) Using microcontact printing, cells can be patterned into well-defined morphologies. (*B*) On disk-like cells CDRs propagate in lateral direction between the cell nucleus and the cell edge (S14 Movie). (*C*) Circular kymograph sampled at the red circle in (*B*). Waves propagating in lateral direction show up as dark stripes. “<“-shaped objects correspond to wave initiation (the green arrow highlights one example) and “>“-shaped objects to wave annihilation. The solid red arrow shows an example of mutual annihilation while the hollow red arrow marks an event in which one pulse survives the collision. (*D*) The apparent high regularity in slopes and frequency of occurrence in *C* is emphasized in an autocorrelation function *c*(Δ*s*, Δ*t*). In this specific example we find propagation velocities of 0.10 µm/s and a typical period of 6 min between two CDR events at the same position (see the cut *c*(Δ*s* = 0, Δ*t*) and *F* for the sample average). The cut *c*(Δ*s*, Δ*t* = 0) emphasizes the dominant number of four DCRs at the same time on this cell. (*E*) A histogram of velocity data obtained from an autocorrelation analysis of 38 cells. The mean velocity is 0.12 (± 0.03) µm/s (± SD). (*F*) A cut through the average correlation function of the same 38 cells at constant position. The mean period between two CDR events at the same position is approximately 6 min. The scale bars in *A* and *B* correspond to 50 µm and 25 µm respectively. See S1 Text, *Materials and Methods* for details regarding the calculation of autocorrelation functions and derived values form these.

We found the propagation velocity of CDRs to be a dynamic quantity on naturally shaped cells. Since we observed the encounter of CDRs with the cell edge or nucleus to crucially influence their dynamics, we where curious how CDRs would behave on cells exhibiting a ring-like region for CDR propagation around the nucleus, i.e., cells of disk-like morphology with centered nuclei. Unfortunately, such morphology is not common for fibroblasts. However, on single cells that happened to have this morphology by chance, we found CDRs orbiting the nucleus with constant velocities (Fig. 5, S13 Movie). Based on this result, we chose to force cells into disk-like morphologies—using microcontact-printing—to realize the simplest possible boundary geometry. On these cells, the nucleus was situated at their center (Fig. 6A&B, S14 Movie). Since CDRs avoided crossing of the nucleus, the resulting path of CDR propagation was quasi one-dimensional with respect to CDR propagation direction and had periodic boundary conditions. On random-shaped cells, CDRs extended over the whole cell surface in some cases. In contrast, on disk-like cells the small region for CDR propagation, i.e., the area between cell nucleus and cell edge, only allowed for waves of a small extent when compared to the cell size (Fig. 6B, S14 Movie). CDRs formed spontaneously on cells of controlled morphologies, without requiring stimulation by growth factors. We verified that these structures indeed corresponded to CDRs based on comparison between them and CDRs formed with PDGF stimulation. Both experimental approaches leaded to CDRs with the same morphological properties (S4 Fig.). In the following we report on a data set of 38 cells. We used kymographs in which we plotted the image intensity along a circle with origin at the cell nucleus versus time to display wave propagation in lateral direction (Fig. 6B&C). We found waves to exhibit remarkably conserved velocities on disk-like cells (Fig. 6C). Wave initiation was followed by expansion of a circular wave that split shortly after formation, which along a line of fixed radius appeared as the propagation of two pulses into both lateral directions. Propagating pulses usually collided head-on with newly formed pulses on their way. Collisions of pulses lead in 79% of the cases to mutual annihilation while in 21% of the cases one pulse survived the collision (Fig. 6C). In other cases CDRs translocated as a whole and therefore appeared in kymographs as two pulses propagating in the same direction, where one pulse closely followed the other.

We segmented kymographs and calculated autocorrelation functions for a systematic quantitative analysis (Fig 6D). Velocities were extracted from autocorrelation functions using an approach based on the Radon transformation (S1 Text, *Materials and Methods*) and from these velocity histograms were calculated. We found a mean propagation velocity of 0.12 (±0.03) µm/s (± SD, Fig. 6E). From averaged autocorrelation functions (S1 Text, *Materials and Methods*) we calculated the temporal correlations at fixed positions. The resulting cut *c*(Δ*s* = 0, Δ*t*) shows a pronounced time interval with negative correlations, i.e., a quiescent time before emergence or passage of the next CDR. The mean period between two successive wave events at one position was 6 min (Fig. 6F). CDRs tended to appear in equidistant positions around the nucleus forming patterns in lateral direction. The cut *c*(Δ*s*, Δ*t* = 0) in Fig. 6F reflects the four-fold rotational symmetry visible in Fig. 6B.

## Discussion and Conclusions

### CDR growth originates from points

Our results regarding typical CDR sizes and lifetimes largely agree with previously reported values [6–8]. However, so far little emphasis has been put on a characterization of the initiation of CDRs. Rather, the early stages of CDRs have been described as “immature” flat and open structures [7]. Early CDRs are reported to consist of punctual short actin filaments that only later coalesce into circular arrays of bundled actin filaments which are then termed “mature” CDRs [8]. While we did not study the details of the actin structure of CDRs in this work, we showed that CDRs can grow as closed mature rings right from the time point of their initiation. Furthermore, we find that the velocity of CDRs is maximal right after initiation and decays with CDR expansion. Our description is therefore in line with the study by Zeng et al, in which they investigated the dynamics of CDR life courses based on cells that were stimulated with PDGF and then fixed [16]. They describe early CDRs as rings with small radii. In their work the time between stimulation and fixation was quantized with a minimal step width of 2.5 min. However, our results show that the dynamics of CDRs varies on a much shorter time scale than minutes and thus requires live cell imaging with frame rates on the order of seconds (Fig. 4, S11 Movie).

### CDRs on cells of uncontrolled morphology lack typical velocities

Peleg et al reported CDR propagation velocities of 2.3 (±0.6) µm/s for closing CDRs [17]. In contrast, the experimentally determined radii as a function of time as published by Zeng et al allow to read off velocities on the order of 0.1 µm/s for opening CDRs [16]. However, it is clear from the results of Zeng et al and especially our detailed analysis that the CDR velocity is a dynamic quantity and we thus cannot state a representative value. Instead, we found that the current velocity of a CDR depends on the ratio of its current size relative to its maximal size. Nevertheless, we found CDRs to exhibit reproducible and temporally constant velocity values on microcontact-printed cells where CDRs are forced into fixed sizes by the boundaries set by cell nucleus and cell edge. On this system we found a mean propagation velocity of 0.12 (±0.03) µm/s (± SD). A visual inspection of supplementary Movie S1 of the work of Peleg et al reveals that the CDRs under investigation in their study exhibit local wavefront instabilities. As shown in Fig 4B, the CDR propagation velocities of partially unstable wavefronts can indeed be much higher than those of stable wavefronts. This explains why the velocities reported by Peleg et al exceed the velocities of the study by Zeng et al and our values by one order of magnitude.

### CDRs can be described as waves in an active medium

The aim of our experimental survey was to systematically investigate CDRs with respect to characteristics of theoretical models of actin waves. Several of the novel phenomena we found for CDRs, such as oscillations, annihilations upon collisions, and spiral waves are well known to occur in active media [19, 20]. The idea to describe CDRs as waves in an active medium was first proposed by Zeng et al [16]. If stimulated locally, their system gave rise to concentric propagating wavefronts. The model explains the reversal of CDR propagation direction as a consequence of a slow kinetics of the inhibitory species. The resulting dynamics is highly asymmetric with respect to the time point of maximal CDR size; in the model, the opening process is much slower than the CDR closing. Our results do not support these outcomes of the model by Zeng et al. In cases where CDR wavefronts did neither reach the cell nucleus nor the cell edge or became unstable, we typically found symmetric profiles of the mean radius with respect to the time point of CDR reversal. The model by Zeng et al predicts the concentration of one of the model’s constituents to govern the maximal CDR size. In contrast we observed that maximal CDR sizes scale with the cell size and are highly variable.

The model by Peleg et al implicates a correlation between local membrane curvature and wavefront velocity, even though the explicitly considered curvature is that in the *xz*- and *yz*-plane [17]. More generally, reaction-diffusion systems often allow for the formulation of an eikonal equation that relates the curvature of the wavefront to its propagation velocity. In contrast, our results show no correlation between local wavefront curvature and propagation velocity. This outcome is puzzling, because we do observe CDRs to propagate as spirals in some cases, and for a persistent spiral pattern there must be a functional relationship between wavefront curvature and velocity [17]. We also found the overall mean curvature not to correlate with the propagation velocity, although we do find the fastest CDR velocity following initiation (smallest radius), and a slowing down as it expands. The overall CDR area seems to govern CDR dynamics.

### CDRs exhibit phenomena resembling characteristics of actin waves in other cell types

While our findings of spiral patterns, oscillations and collision annihilation are novel for CDRs, these phenomena have been described in the scope of actin waves in different cell types previously. Numerous theoretical models have been suggested that capture these behaviors [4, 5, 20–23]. Prominent examples of actin waves organizing into spirals have been reported for *D. discoideum.* The actin waves in this unicellular slime mold act to establish a leading front in *D. discoideum*‘s migration strategy [24–25]. In the process of recovery from actin depolymerization, actin waves in *D. discoideum* form closed, ring-like structures that resemble CDRs in shape and also the propagation velocities are in the same range as the velocities we found for CDRs [25]. Actin waves propagating with similar velocities are also found in neutrophils [4]. In contrast to CDRs, waves in *D. discoideum* and neutrophils are not associated with vertical projections at the dorsal cell side. Waves of phosphatidylinositol(3,4,5)-trisphosphate, that play a major role in the organization of actin waves in *D. discoideum*, explicitly form at the basal side of these cells [26]. In the wave mechanism underlying CDRs, the involvement of a protein species that selectively aggregates at the dorsal cell side is an attractive assumption. In the model by Peleg et al, the selectivity is provided by the deformation of the cell surface and the subsequent accumulation of curvature-sensitive proteins.

### CDR dynamics is governed by the limited availability of some reactive species

We propose that there must be two contributions to the dynamics of wavefront velocities of CDRs. One stems from a reaction-diffusion system that creates waves and makes them propagate. In most cases, however, the actually visible dynamics is dominated by another contribution due to limited resources, which masks the inherent dynamics of the reaction-diffusion system. One could think of several possible effectors, among them membrane tension or limited availability of some reactive protein species. This picture is consistent with our finding of uniform propagation velocities of CDRs on micro-patterned cells. On these cells the CDR size is limited to the space available between cell nucleus and cell edge. Edgar and Bennett observed that CDRs avoid the cell nucleus before and pointed to a possible role of intermediate filaments in this phenomenon [27]. Regardless of the underlying mechanism, our experimental design ensures that CDRs do not change their size upon propagation. This implies that for propagating CDRs on micro-patterned cells the membrane area remains constant. Also the amount of proteins incorporated in a CDR should be constant. With this spatial confinement we also limit the curvature of wavefronts to a specific value. Given the homogeneity of the medium for propagation in lateral direction on disk-like cells, the exhibited constant velocities might follow as a natural consequence. We therefore found a way to allow investigations of the wave propagation without disturbing effects due to limited species or membrane area.

As discussed in Allard and Mogilner’s work, travelling waves provide an economic way to support cell motility in situations of limited resources of, e.g., the actin regulating protein VASP or actin itself [2]. Since we do observe correlation between CDR stalling and drastic actin depletion (Fig. 3A, S6 Movie), the limiting species could indeed be actin.

Holmes et al investigated the effects of limited resources of regulatory proteins on the propagation of actin waves in a theoretical study [28]. Their reaction-diffusion system produces waves that stall close to the boundary of the system, a phenomenon, which is studied in detail in a succeeding work and called “wave pinning” [29]. Interestingly, the pinned waves in their study exhibit local oscillations around their average position much like the stalled CDRs close to cell edges do in our study. In a different scenario, their model produces waves that slow down and reverse their propagation direction upon approach of boundaries. The effects due to limited resources are thus not only able to explain the slowing down of propagating waves, but also their reversal.

In this picture, the closing of CDRs does not require a contractile element as proposed by Hoon et al [7]. Rather, just like the closed circular shape is a natural consequence of a wave in an active medium that was stimulated in one point, closing back of the wave to one point is a natural consequence of the reflection of these waves from boundaries such as the cell edge or the cell nucleus. This idea is also in accord with our finding that CDR geometries tend to mimic the cell shape.

### Mutual CDR annihilation suggests the existence of an invisible protein field preceding actin waves

In their current review, Allard and Mogilner call the mutual annihilation of colliding wavefronts a “signature of excitation waves” [2]. Zeng et al hypothesized before that colliding CDRs would mutually annihilate [16]. Their idea was thereby supported by the fact that they never found intersecting CDRs on fixed cells. Our results partially agree with this picture. We indeed observed mutual annihilation of wavefronts when CDRs happened to collide on random-shaped cells. However, on cells on microcontact printed substrates we also found collisions in which one of the two wavefronts survived. Even though this behavior might seem to contradict an active medium description at first glance, theoretical work by Argentina et al has shown that the FitzHugh-Nagumo model supports crossing of wave pulses [30]. The FitzHugh-Nagumo model is a prototype model of an active medium that supports local oscillations and wave propagation including expanding rings exhibiting instabilities [31]. The model however fails to reproduce our observation of waves that annihilate before they actually make contact, regardless of whether the visible wave corresponds to the activating or the inhibiting species. However, this observation strongly implies that a wave of an actin regulator precedes the visible actin wavefronts. The molecular identification of this wave and a detailed investigation of CDR collisions are future challenges and will add fundamentally to our understanding of CDRs.

### CDR oscillations support an active medium description

Contrary to statements by Buccione et al [6] and Itoh and Hasegawa [8] that CDRs would only form once, our results indicate that CDRs have a strong tendency to reappear periodically with typical intervals of 5–6 minutes. Especially for cells on microcontact printed substrates, repeated formation of CDRs was the rule rather than the exception. However, the procedure for CDR stimulation that is normally followed in the literature relies on addition of growth factors, such as PDGF, to the cell medium. For cells, this means a sudden disturbance of their biochemical state. In contrast, it was our focus to study cells over long times under constant biochemical conditions. We thus refrained from growth factor stimulation. Instead, we relied on the growth factors contained in FBS, which is a standard constituent of cell culture medium. This limits our knowledge about the details of the biochemical trigger of wave formation but enabled long-term experiments.

If we assume that under our regular experimental conditions cells were stimulated by growth factors contained in the FBS, we can formulate two hypotheses that account for the observed oscillatory reappearances of CDRs. The first is based on the fact that CDRs internalize the receptors that lead to their stimulation via endocytosis [32]. In this picture, growth factor stimulation would lead to CDR formation, which goes along with receptor internalization. As soon as the internalized receptors were renewed, the permanent exposure to growth factors in the cell medium again would lead to CDR formation and so on. Under this assumption, the observed periods between two succeeding CDRs would correspond to the time scale of receptor renewal. In this picture, however, cells stimulated with PDGF should also continuously form re-appearing CDRs, which is not reported in the literature [6, 8, 10].

The second hypothesis is based on characteristics of active media. From a theoretical perspective, wave propagation and oscillatory states are closely related phenomena and several models support both behaviors. Classical systems like the FitzHugh-Nagumo model respond with oscillatory behavior to stimuli, if a limit cycle trajectory in phase space is reached upon excitation. In this case, only a single stimulation would be needed to trigger oscillatory CDR formation. The period between two successive CDRs would correspond to the recovery time of the active medium.

We cannot make definite statements about whether repeated CDR formations were due to repeated stimulations or due to a biochemical state of cells that does not need continuous stimulation to repeatedly trigger waves. Indeed, experiments under FBS-free conditions indicated that cells are even capable of spontaneous CDR formation in the absence of growth factors (see S1 Text, *SI Results* and Fig. IV therein). Therefore, one might speculate that CDRs cannot only form due to growth factor stimulation but also spontaneously. Spontaneous formation of actin waves has indeed been described in *D. discoideum* and theoretically investigated by Whitelam et al [21]. Recent work by Wu et al shows that coupling of two oscillators can account for spatio-dynamic actin patterns in mast cells, providing an attractive line of thought for the interpretation of patterns we observe on disk-like cells (see *c*(Δ*s*, Δ*t* = 0) in Fig. 6D) and future experiments [5].

## Concluding Remarks and Outlook

We showed in this work that CDRs are a phenomenon that is in agreement with recent theoretical models of actin waves. The apparently complex dynamics of CDRs on cells of uncontrolled morphology can be understood if we assume that a limited resource influences their dynamics. The impact of this limiting resource scales with CDR size. Consequently, CDRs of fixed sizes propagated with constant velocities and regular patterns of reappearance.

Most cell types attain irregular shapes when adhering to substrates. We showed in this work that actin-membrane waves strongly depend on boundary geometry, which complicates quantitative studies of these waves. Furthermore, the propagation velocities of actin waves are of the same order of magnitude as the velocities of changes of cell shape, e.g., the velocities of lamellipodia protrusion [33–35]. This raises questions for the appropriate frame of reference for wave propagation on the time-dependent cell morphology, even though there has been progress along these lines recently [20]. On cells adhering to symmetrically shaped protein patches controlling their morphology, waves propagate in a medium that is homogeneous in lateral direction. Especially, there are no boundaries in this direction. We thus propose that microcontact printing provides ideal means for the study of protein waves in cells. This holds especially true, because it allows for a direct comparability with theoretical studies, in which concepts like shapes of reduced complexity and periodic boundary conditions are often used [21, 23, 36].

One of the most prominent features of CDRs is their ability to strongly deform the cell surface [6, 11, 12]. For a full understanding, their three-dimensional nature must thus be taken into account. Peleg et al took the first steps towards such a three-dimensional modeling [17]. It will be a future task to characterize protein dynamics in three dimensions also experimentally. For this, the quick dynamics of CDRs and their large extension into vertical direction are the major challenges.

The three-dimensional nature of CDRs is also the main feature differentiating them from other kinds of actin waves like, e.g., the spiraling waves on the basal side of *D. discoideum* [37] or lateral membrane waves along cells spreading on planar substrates [38]. However, all of these waves are observed on cells of quasi-circular morphology, which highlights the relevance that symmetry has for the formation of well-observable actin waves of unperturbed propagation.

## Materials and Methods

### Cell culture

We used NIH 3T3 (ATCC CRL1658) fibroblasts for experiments involving microcontact printing. The NIH 3T3 cell culture was a gift from Louis Lim (Institute of Molecular and Cell Biology, ASTAR Singapore). For long-term experiments we employed a genetically modified variant termed NIH 3T3 X2 [39]. Compared with standard 3T3 cells, an enlarged fraction of X2 cells exhibited CDR formation. Cells were grown under standard conditions in Dulbecco’s MEM containing 3.7 g/L NaHCO_3_, 4.5 g/L D-Glucose (Biochrom), 100 µg/ml Penicillin/Streptomycin (PAA), and 10% Fetal Bovine Serum (Biochrom). Cells were split at 80% confluency using Trypsin/EDTA (Biochrom). Cells were mycoplasma free.

### Microcontact Printing

We followed the protocol by Théry et al for preparation of microcontact-printed substrates with minor modifications [40]. We created a silicon master containing disk structures using deep reactive ion etching. From this master, polydimethylsiloxan (PDMS, Sylgard 184 silicone elastomere) negatives were cast that served as stamps for microcontact printing.

We used a Kinpen 11 (Neoplas Control) for activation of glass substrates under sterile working conditions. The glass substrates were then stamped with human fibronectin (Roche) that was allowed to adhere to PDMS stamps before. After this the substrates were treated with PLL(20)-g-[3, 5]-PEG(2) (SuSoS Surface Technology) to prevent cell adhesion outside of fibronectin-printed areas. We used disks with an area of 3000 (µm)^2^.

### Sample preparation and imaging

For imaging of random-shaped cells, we plated cells at intermediate confluency on plasma-treated glass bottom dishes. After cells were fully spread, we washed the samples with Phosphate Buffer Saline (PBS) and added fresh DMEM. Imaging was then started and typically lasted several hours. For experiments on cells on fibronectin patterns we plated cells on microcontact-printed substrates and allowed them to adhere for 20 minutes. Samples were then thoroughly rinsed with PBS and supplied with fresh DMEM. Imaging was started thereafter and lasted typically one hour. For fluorescence microscopy cells were transfected with the actin binding peptide pLifeAct–TagGFP2. We used human-platelet derived growth factor BB (hPDGF-BB, Cell Signaling Technology) at a concentration of 30 ng/ml in serum-free DMEM in experiments with growth factor stimulation on naturally shaped cells. For experiments with cells on disk-like protein patches much lower concentrations were needed (1 ng/ml).

Live cell imaging was performed using a Zeiss Axio Oberver.Z1 equipped with a Zeiss incubation system consisting of Heating Unit XL S, Temp Module S, a Pecon Heating Insert P S1 and a CO_2_ Module S. All experiments were carried out at a temperature of 37°C and 5% CO_2_. For low-resolution and low-magnification imaging, a Zeiss Achro Plan 10x with a numerical aperture of 0.25 was used with a 1.6x optovar lens. For intermediate resolution and magnification we utilized a Zeiss Plan Apochromat 40x with a numerical aperture of 0.95. A Zeiss AxioCam MRm was employed for image acquisition in conjunction with Zeiss AxioVision Software. For fluorescence imaging, samples were illuminated with a Zeiss HXP120 mercury lamp, a 488 nm BrightLine HC filter (AHF Analysetechnik) and a Zeiss 76 HE reflector filter set.

### Image processing and contour analysis

All image and data processing was carried out in MATLAB2012b (The Mathworks) and FIJI [41] using custom-written routines.

For visualization of spiral wave patterns (Fig. 3C), we segmented time-lapse sequences via visually determined gray value thresholds and subsequent manual removal of artifacts (S3A Fig.). The resulting binary time-lapse sequences (S3B Fig.) were then displayed using a 3-d viewer included in FIJI, following [25]. In 3-d views the background color black was set to transparent and the white pixels were displayed as volumetric objects. The surfaces of these objects were then rendered in *x-y-t*-space (S3C Fig.). S8 Movie was produced using the ZEN 2012 Software (Zeiss).

We used active contours to track the wavefronts of CDRs. Comparing images of fluorescence microscopy and images of phase contrast microscopy, we found that maximal actin concentrations corresponded to minima in phase contrast (S2 Fig.). We thus used phase contrast imaging to track CDRs and could therefore avoid issues such as photobleaching and phototoxicity. For the implementation of the active contour algorithms we followed Xu and Prince [42] and created a graphical user interface in MATLAB allowing user control and interaction. A rough initial guess of the position of the contour was based on a user input in form of a drawing. The program then let the contour converge towards the minimum of image intensity, which was the position of the CDR wavefront. For all following frames of a time-lapse sequence the program used the final state of the last iteration as the initial guess for the next frame etc. To exclude the bending energy of active contours to affect measurements of contour curvature, we performed one final relaxation step. We implemented this relaxation in the following way. For each contour point a computer program sampled an image intensity profile in normal direction of the contour. The profile was then locally approximated with a cubic function. The position of the contour point was then changed towards the global minimum of this cubic function. The data we obtained from our ruffle tracking routines were sets of contour points. The distance between adjacent contour points was 0.8 µm, which matches the resolution limit of the 10x objective. We performed a final smoothing of contours with a Sobel kernel with a width of the order of our resolution limit.

The individual contour points were the basis for calculation of contour velocities. For each contour point we calculated rays in normal direction with respect to the local contour shape. We then determined the intersection point of this ray with the contour of the next frame. We thereby interpolated linearly between the contour points of the contour of the next frame. The distance between contour point and intersection point divided by the time interval between two frames corresponded to the local velocity.

## Supporting Information

S1 Text PDF containing conceptual details of the contour representation of CDRs, mathematical details regarding the fit in Fig. 4F, the calculation of autocorrelation functions, the results of a correlation analysis between contour velocity and curvature, and experiments on the correlation between FBS concentration and the frequency of occurrence of CDRs. This document contains Fig. I, II, II, and IV.
(PDF)

S1 Fig Time-lapse sequence of NIH 3T3 X2 cells stimulated with PDGF. Cells were incubated in serum-free DMEM for two hours prior to the experiment. Serum-free DMEM containing 30 ng/ml PDGF was then added to the cells directly before imaging. CDR coverage of cells is maximal 10–20 min after stimulation. We highlighted examples of CDRs on two cells with red arrows. Scale bar: 25 µm.
(EPS)

S2 Fig Maxima in actin concentration correspond to minima in phase contrast intensity. Overlay of phase contrast and pLifeAct–TagGFP2 fluorescence channels of a micrograph showing CDRs. The scale bar corresponds to 25 µm.
(TIF)

S3 Fig Processing steps for *x*-*y*-*t*-visualization of CDR spiral waves. (*A-B*) Application of a visually determined gray value threshold turns phase contrast images (*A*) into binary images (*B*). In (*A*) the original phase contrast image is shown as the gray channel. The overlaid red channel is the binarized image (*B*). (*C*) A 3-d visualization of binary images as an iso-surface of image intensity shows the spatio-temporal dynamics of spiraling CDRs.
(PDF)

S4 Fig Morphological comparison of CDRs on cells of controlled shape due to PDGF stimulation and due to spontaneous formation in FBS-containing DMEM. The CDRs formed in response to growth factor stimulation correspond to the CDRs formed spontaneously in FBS-containing cell medium. Cells were washed thoroughly with PBS and then kept in serum-free media prior to addition of PDGF (1 ng/ml in serum-free DMEM).
(PDF)

S1 Movie Oscillatory-reappearing CDRs on spatially narrow regions. The movie underlying Fig. 1A.
(MP4)

S2 Movie CDR exhibiting expansion and propagation while maintaining roughly circular shape. Note that the initial phase of CDR formation was not captured. The movie underlying Fig. 1B.
(MP4)

S3 Movie CDR of high anisotropy strongly mimicking the cell shape. The movie underlying Fig. 1C.
(MP4)

S4 Movie CDR of large extension covering almost the entire cell exhibiting CDR stalling and division. The movie underlying Fig. 1D.
(MP4)

S5 Movie Oscillatory Reappearance of Small CDRs. The full movie underlying Fig. 2A–C.
(MP4)

S6 Movie Stalling of CDRs Correlates with Actin Depletion. The full movie of which a still is shown in Fig. 3A.
(MP4)

S7 Movie Spiraling CDR. The full movie underlying Fig. 3B&C. The Length of the movie exceeds the 9 min interval shown in Fig. 3B. Negative time stamps correspond to times before the first frame shown in this figure.
(MP4)

S8 Movie Spiraling CDR in 3-d Visualization. An animated 3-d rotation of the object shown in Fig. 3C around the *x*-axis. Please note that Fig. 3C shows the object as an iso-surface while this movie uses a maximal intensity projection, which was found more suitable for this case. In the movie, the *x*-axis is shown in red, the *y*-axis in green and the *t*-axis in blue.
(MOV)

S9 Movie Dividing CDRs. The movie underlying Fig. 3D. Negative time stamps correspond to frames before the first frame shown in Fig. 3D.
(MP4)

S10 Movie CDR Collisions. The full movie underlying Fig. 3E&F. The length of the movie exceeds the 0.9 min shown in Fig. 3E&I. Negative time stamps correspond to times before the first frame shown in this figure.
(MP4)

S11 Movie Wavefront Dynamics of CDRs. The full movie underlying Fig. 4A–C.
(MP4)

S12 Movie Contour Tracking of CDRs. A CDR and its contour representation. Note that this movie serves to backup Fig. 4D&E. Therefore, the contour of the second CDR visible in the movie is not shown. It was, however, included in the dataset underlying Fig. 4F.
(MP4)

S13 Movie CDR orbiting the nucleus of a disk-shaped cell. The full movie underlying the time-lapse sequence shown in Fig. 5A. Note that this cell is not plated on a protein patch but has a disk-morphology by chance. Negative time stamps correspond to frames before the first frame shown in Fig. 5A.
(MP4)

S14 Movie Uniform Propagation of CDRs on Disk-Like Cells. The full movie underlying Fig. 6B–D.
(MP4)
