# Correction: Dynamics of Actin Waves on Patterned Substrates: A Quantitative Analysis of Circular Dorsal Ruffles

**DOI:** 10.1371/journal.pone.0119746

**Published:** 2015-03-25

**Authors:** The PLOS ONE Staff

Due to a typesetting error, Fig. 2 is missing an essential variable (the Greek τ “tau”) in three places. The publisher apologizes for the error. Please view the complete, correct Fig. 2 here.

**Fig 2:**
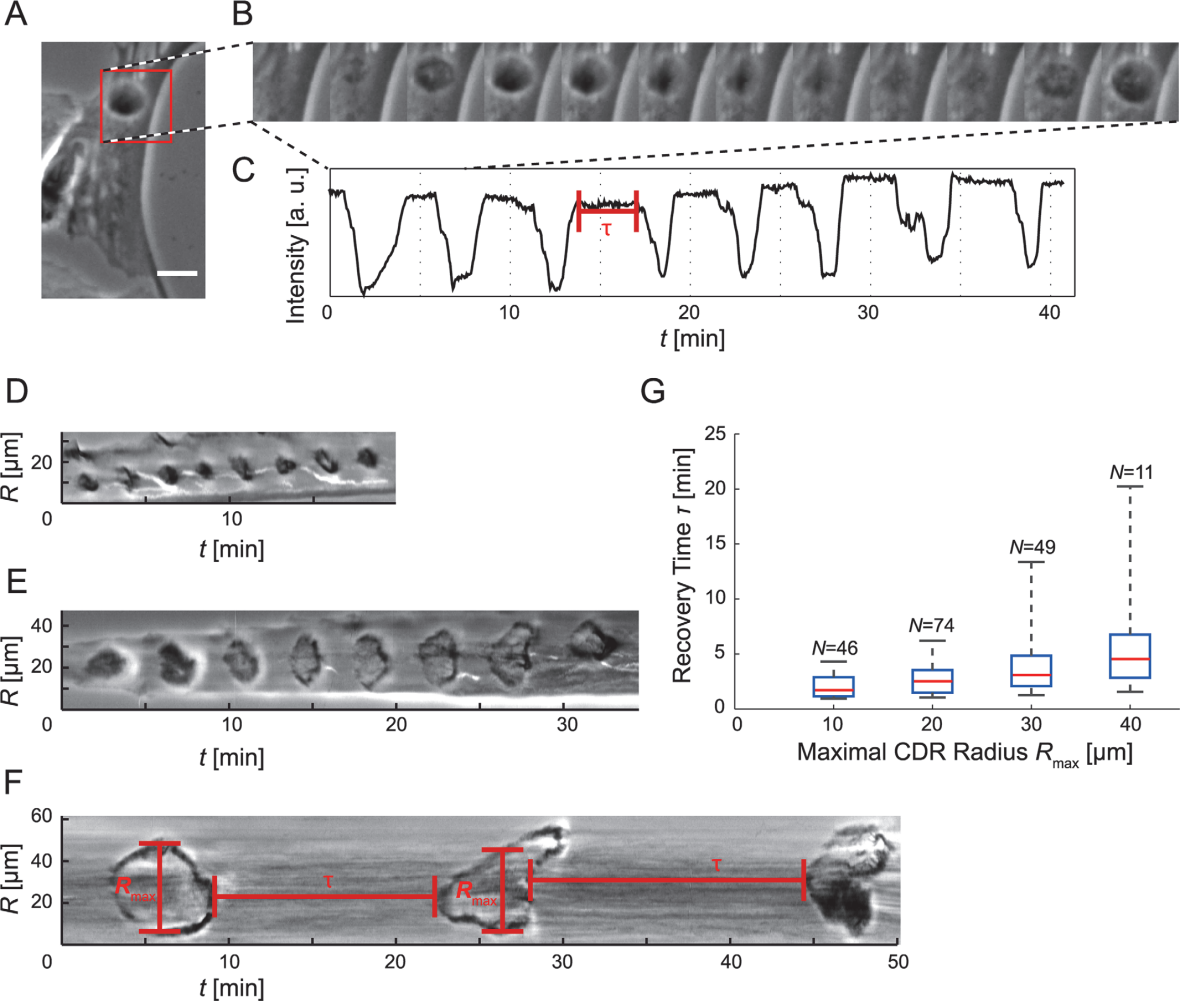
Oscillatory reappearing CDRs. (*A*) CDRs under spatial confinement exhibit oscillatory patterns of pulsating re-appearance (scale bar: 25 μm, full sequence: S5 Movie). (*B*) Stills from the region of interest highlighted red in the time-lapse sequence *A* (Δ*t* = 36 s). (*C*) A plot of the minimal intensity value of the ROI in *A* as a function of time shows CDR events as negative peaks and CDR-free periods, corresponding to the recovery time τ, as plateaus of high intensity. The ROI was smoothed with a Gaussian with *σ* = 2 μm prior to intensity sampling. (*D-F*) Kymographs of CDRs taken along lines crossing CDR origins (see Fig. 4*A*for illustration) show both the recovery time τ between successive events and their radial extension *R*_max_ (cells not shown). (*G*) The recovery times increase with CDR size. The data was binned in *R*_max_-direction (box width: 10 μm) and plotted as boxes with whiskers (red lines: median, upper box edge: 75th percentile, lower box edge: 25th percentile). *N* values denote the number of observations. Note that oscillatory behavior was rare for large CDRs.
