# Supplementary material for: Dynamics of Actin Waves on Patterned Substrates: A Quantitative Analysis of Circular Dorsal Ruffles
Source: PLoS One. 2015 Jan 9;10(1):e0115857. doi: 10.1371/journal.pone.0115857 (PMC4289068; doi:10.1371/journal.pone.0115857)
Supplement: S1 Text — This document contains Fig. I, II, II, and IV. (PDF) [file pone.0115857.s001.pdf]

Dynamics of Dorsal Actin Waves (Circular Dorsal Ruffles) - Supporting Information -

Erik Bernitt[a], Cheng Gee Koh[b], Nir Gov[c], Hans-Günther Döbereiner[a]

Author Affiliation:
[a]Institut für Biophysik, Universität Bremen, Bremen 28359, Germany
[b]School of Biological Sciences, Nanyang Technological University, Singapore 639798, Singapore
[c]Department of Chemical Physics, Weizmann Institute of Science, Rehovot 7610001, Israel

Corresponding Author:
Erik Bernitt
Institut für Biophysik
Universität Bremen
Otto Hahn Allee
28359 Bremen
Tel. +49 421 218 62308
Email: ebernitt@uni-bremen.de

SI Materials and Methods

Contour-Derived Measures

Contours are sets of N discrete points (x_i,y_i) with a certain order and spacing between neighbouring contour points. We chose a spacing of 0.8 µm, which corresponds to the resolution of the 10x objective (Fig. II*A*-*B*).

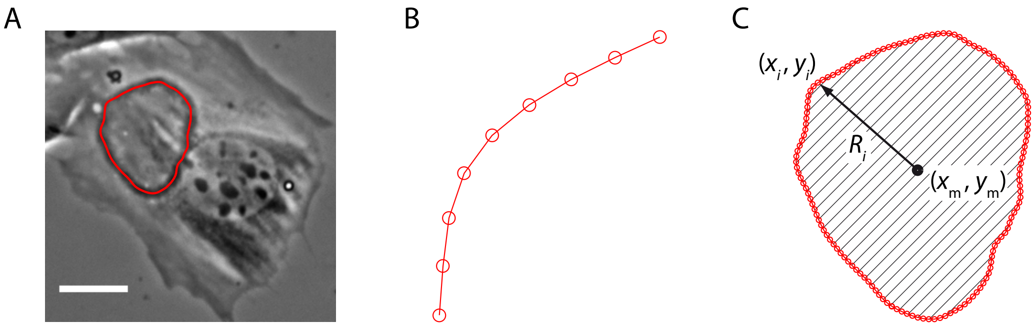

Fig. II Representation of CDRs as contours and definition of contour properties. *A*: CDR and its contour representation. The scale bar corresponds to 25 µm. *B*: a close-up view on the contour shows the shaping contour points. *C*: The coordinates (x_i,y_i) of every contour point have a distance R_i from the center of gravity (x_{m}, y_{m}) of the contour. The shaded region represents the area A covered by the CDR.

For calculation of the mean radius R of contours and the area A enclosed by contours (Fig. II), we proceeded as follows. We first calculated the center of gravity (x_{m},y_{m}) of the contours according to

$$x_{\mathrm{m}} = \frac{1}{N}\sum_i^N x_i \qquad \text{and} \qquad y_{\mathrm{m}} = \frac{1}{N}\sum_i^N y_i$$

and from this the radii

$$R_i = \sqrt{(x_i - x_{\mathrm{m}})^2 + (y_i - y_{\mathrm{m}})^2}$$

which finally yields the mean radius

$$R = \frac{1}{N}\sum_i^N R_i.$$

We calculated the area enclosed by a contour approximating Stoke's theorem according to

$$A = \frac{1}{2}\oint (x\mathrm{d}y - y\mathrm{d}x) \approx \frac{1}{2}\sum_i^N (x_i(\Delta y)_i - y_i(\Delta x)_i)\,.$$

where $(\Delta x)_i$ and $(\Delta y)_i$ are the distances of two neighbouring contour points in x- and y-direction respectively.

Fitting the Velocity Data as a Function of the Normalized Area

Based on kymograph analysis, we knew that CDRs, which did not collide with the cell edge or the nucleus, showed a quadratic radius evolution with time (Fig. 4*B*) and could therefore be fitted with the following parabola:

$$R(t) = R_{\mathrm{max}} - at^2. \tag{S1}$$

Here, R_{max} is the maximal radius, a the fitting parameter, and t the ruffle life time, measured with respect to the time point of maximal radius.

If we assume that CDRs are perfectly circular, the area covered by a CDR is given by

$$A(t) = \pi R(t)^2.$$

With the definitions

$$\widetilde{A}(t) = \frac{A(t)}{\pi R_{\mathrm{max}}^2}\ ,\ v_{\mathrm{n}}(t) = \frac{\mathrm{d}R(t)}{\mathrm{d}t}\ ,\ \text{and}\ \alpha = \frac{1}{4aR_{\mathrm{max}}}$$

a self-consistent relation for the scaled CDR area can be written as

$$\widetilde{A}(v_{\mathrm{n}}(t)) - 1 = -2\alpha v_{\mathrm{n}}(t)^2 + \alpha^2 v_{\mathrm{n}}(t)^4. \tag{S2}$$

This function was used to fit the data in Fig. 4*F*.

Kymograph Segmentation and Calculation of Autocorrelation Functions

We segmented phase contrast kymographs of CDRs that rotated around the cell nucleus using visually-determined gray value thresholds. From this we obtained binary kymographs, in which a value of $B_{s,t} = 1$ and $B_{s,t} = 0$ corresponded to time and space coordinates with and without CDR respectively. Note that the quantities propagation velocity and period of ruffling events do not depend on the exact height of gray value thresholds. For the calculation of correlation functions we took advantage of the periodicity of kymographs in space by replication of the kymograph in negative and positive direction in space. From these replicated kymographs we calculated correlation functions based on the formula

$$c_{\Delta s,\Delta t} = \frac{1}{N_s N_t - |\Delta s \Delta t|} \sum_{s=-\left(\frac{N_s}{2}-\Delta s-1\right)}^{+\frac{N_s}{2}-\Delta s-1} \sum_{t=0}^{N_t-\Delta t-1} \left(B_{s+\Delta s,t+\Delta t} - \bar{B}\right) \cdot \left(B_{s,t} - \bar{B}\right). \tag{S3}$$

Here, N_t and N_s are the data points in spatial and temporal direction respectively. $\bar{B}$ is the mean value of the binarized kymograph.

Determining Velocities from Autocorrelation Functions via Radon Transformation

Signals of constant velocity in kymographs yield stripe-signatures in autocorrelation functions of these kymographs (Fig. 6*C* and *D*). We used the Radon transformation for the quantification of the slope $\frac{\Delta s}{\Delta t}$ of stripe patterns in autocorrelation functions.

In image analysis, the Radon transformation is an integral transformation that projects an image $I(x, y)$ along parallel rays on an axis with coordinate ρ. The ρ-axis has an angle Θ with respect to the x-axis of the image. The Radon transform can be calculated as

$$g(\Theta, \rho) = \int_{-\infty}^{+\infty} \int_{-\infty}^{+\infty} I(x, y)\delta(x\cos(\Theta) + y\sin(\Theta) - \rho)\mathrm{d}x\mathrm{d}y, \tag{S4}$$

where δ corresponds to Dirac's distribution [1]. The Radon transform of an image containing stripe patterns shows strong variation in ρ-direction at an angle that corresponds to the slope of the stripes (Fig. III). Note that ρ itself has no physically meaningful units. We used an implementation of the Radon transformation included in MATLAB's Image Processing Toolbox (Vers. R2012). All velocity data plotted in Fig. 6*E* in the main text were calculated as described above.

Averaged Autocorrelation Functions

The height of the threshold used for segmentation influenced the width of structures in binary kymographs. It therefore also determined the height of peaks of the autocorrelation function. For the calculation of velocities and periods only the position of peaks and not their height was determining. For a calculation of an averaged correlation function of a set of kymographs however, we had to assure

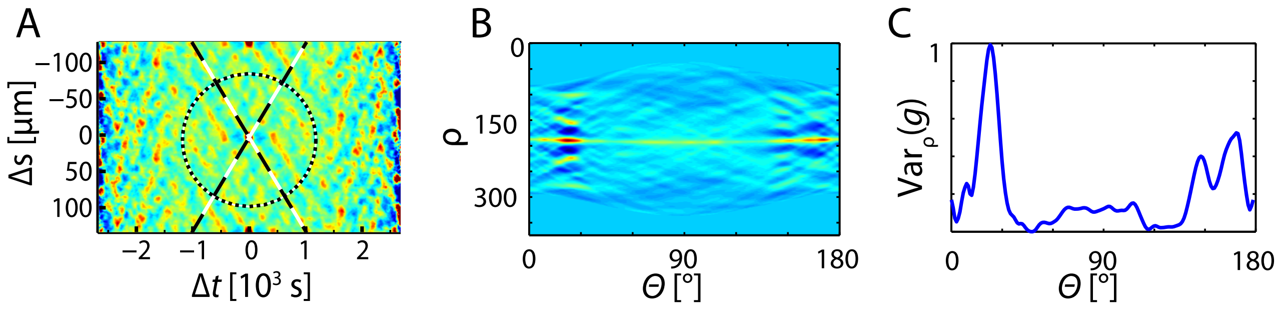

Fig. III Velocity measurements based on the Radon transformation. *A*: Example of a correlation function of a kymograph exhibiting stripe patterns. The dashed lines have a slope of $v = \Delta s/\Delta t$ as determined by the Radon transformation. *B*: Radon transform $g(\Theta, \rho)$ of the circular ROI highlighted by the dotted line in *A*. In this example, the Radon transform has the strongest variation in ρ-direction at Θ=22 ° (*C*), corresponding to v =0.13 µm s^{-1}. The variance plotted in *C* was normalized to unity.

that all kymographs contributed equally. For this, we binarized the autocorrelation function of each kymograph according to

$$\widetilde{c}_{\Delta s,\Delta t} = \begin{cases} 1 & \text{if } c_{\Delta s,\Delta t} > 0 \\ -1 & \text{if } c_{\Delta s,\Delta t} \leq 0 \end{cases}.$$

From these binarized autocorrelation functions we calculated one averaged autocorrelation function. The cut $\widetilde{c}_{\Delta s=0,\Delta t}$ is plotted in Fig. 6*F* in the main text.

SI Results

Velocity and Curvature are Uncorrelated

We determined the local contour curvature by locally fitting cubic splines to contours. From the fit parameters we derived the curvature in an analytic fashion. We analyzed our data for a correlation between local curvature and local velocity. For this we used the same data set underlying Fig. 4*F* in the main text. Since the number of data points exceeded 200,000 a scatter plot appeared too crowded and was no suitable option for display. We thus employed a histogram plot in which we counted the occurrences of data points in rectangular bins of regular width in curvature and velocity. Since contours of large radii have a lower average curvature but contain, due to their large size, more contour points they dominate the data set in terms of numbers. We thus displayed the counts in the histogram shown in Fig. I logarithmically. The plot reveals no correlation between local contour velocity and the local curvature of contours.

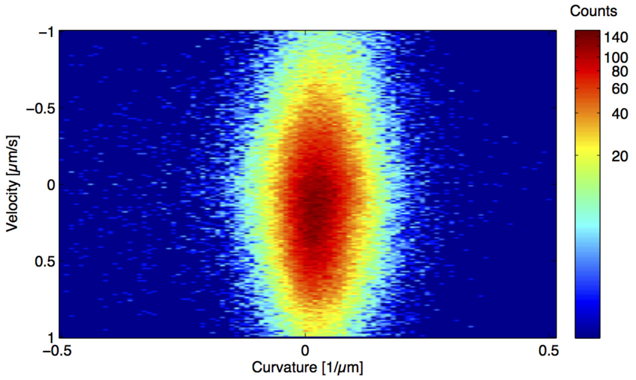

Fig. I Histogram scatter plot of local curvature and local velocity of CDR contours. The counts are displayed color-coded. A logarithmic scale was chosen to avoid over-representation of large contours.

FBS is not Required for CDRs but Enhances the Rate of CDR Formation

We tested how the FBS concentration influences the fraction of cells that show CDRs. For this we used NIH 3T3 X2 cells that were plated 24 hours prior to experiments. Directly before experiments, we added fresh and FBS-free DMEM and imaged cells for 2 hours. We then added FBS to the dish, attaining a final concentration of 10% FBS and imaged the same cells for another 2 hours. We split our time-lapse movies into parts of 30 minutes and counted the fraction of cells that exhibited CDRs in that interval. A significantly lower number of cells exhibited CDRs in FBS-free conditions (Fig. IV). The total number of cells imaged was 1119. We verified the significance of the difference in median values using a two-sided Mann–Whitney U test at a level of $\alpha = 5\%$.

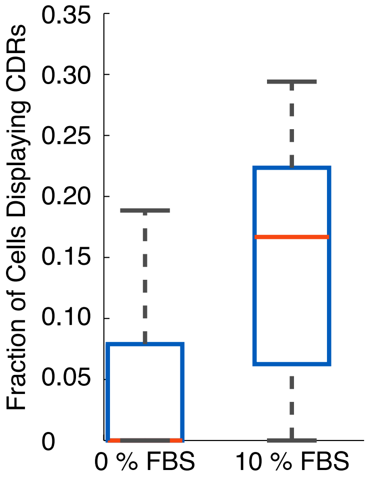

Fig. IV Box and whisker plot showing the effect of FBS on the fraction of cells with CDRs. Under FBS-free conditions, a low fraction of X2 cells exhibits spontaneous CDR formation. However, the median fraction is zero. In FBS-containing DMEM, a median fraction of 0.17 X2 cells exhibits CDR formation. The difference in median values is statistically significant at a level of $\alpha = 5\%$. Whiskers denote minimal and maximal sample values.

References

[1] Gonzales RC, Woods RE (2008) Digital Image Processing. Pearson Prentice Hall. pp. 368-374.
