# Supplementary figures and images for: Dynamics of Actin Waves on Patterned Substrates: A Quantitative Analysis of Circular Dorsal Ruffles
Source: PLoS One. 2015 Jan 9;10(1):e0115857. doi: 10.1371/journal.pone.0115857 (PMC4289068; doi:10.1371/journal.pone.0115857)

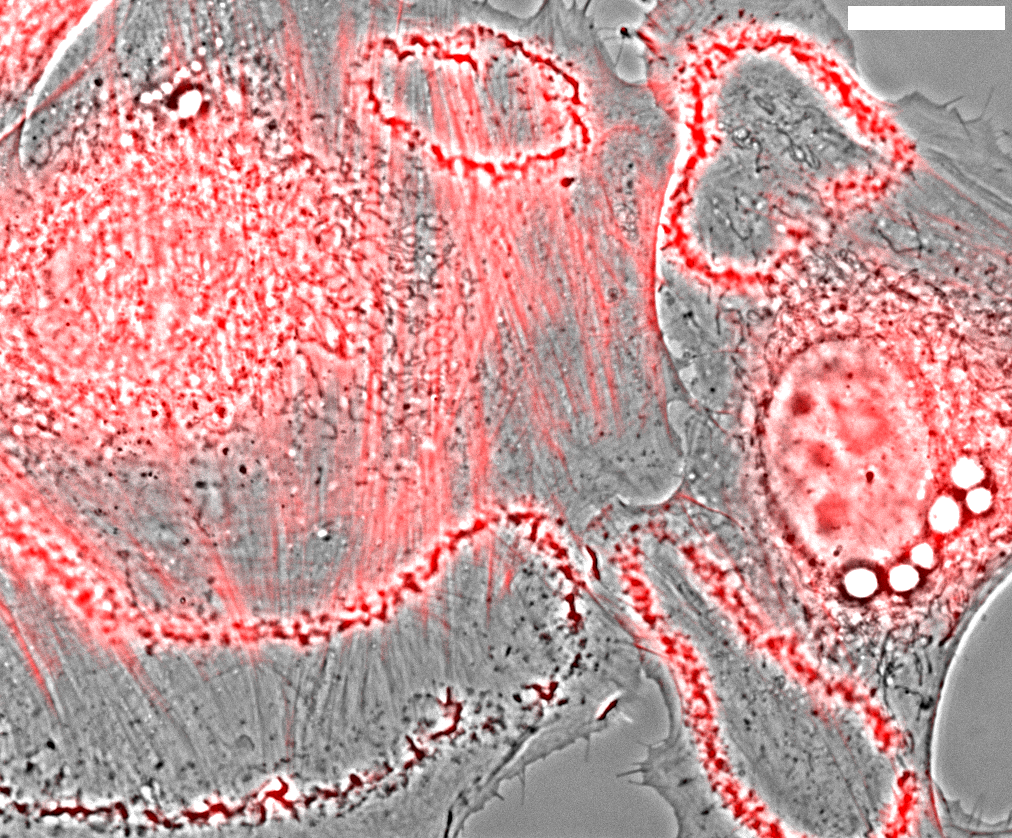

Supplement: S2 Fig — Overlay of phase contrast and pLifeAct–TagGFP2 fluorescence channels of a micrograph showing CDRs. The scale bar corresponds to 25 µm. (TIF) [file pone.0115857.s003.tif]

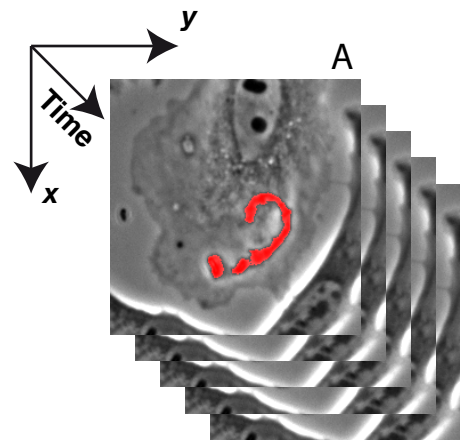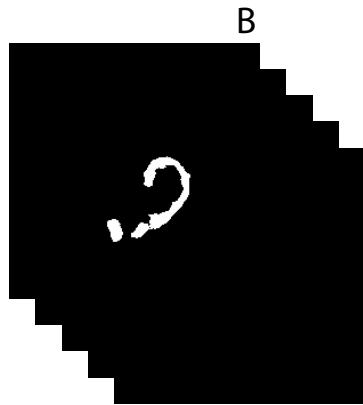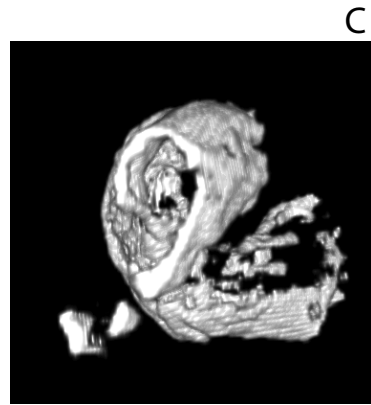

Supplement: S3 Fig — (A-B) Application of a visually determined gray value threshold turns phase contrast images (A) into binary images (B). In (A) the original phase contrast image is shown as the gray channel. The overlaid red channel is the binarized image (B). (C) A 3-d visualization of binary images as an iso-surface of image intensity shows the spatio-temporal dynamics of spiraling CDRs. (PDF) [file pone.0115857.s004.pdf]

+ PDGF

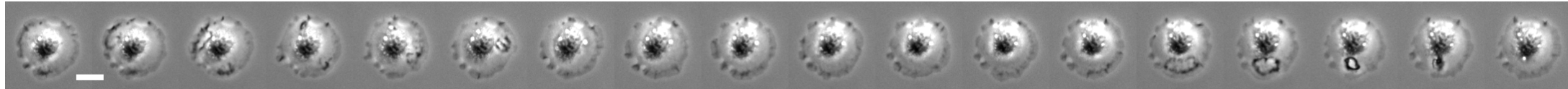

0 min

1.8 min

3.6 min

5.4 min

7.2 min

9.0 min

10.8 min

12.6 min

14.4 min

+ FBS

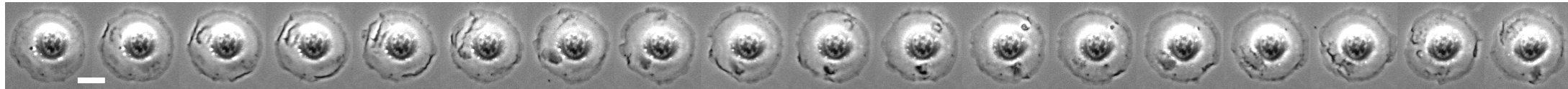

Supplement: S4 Fig — The CDRs formed in response to growth factor stimulation correspond to the CDRs formed spontaneously in FBS-containing cell medium. Cells were washed thoroughly with PBS and then kept in serum-free media prior to addition of PDGF (1 ng/ml in serum-free DMEM). (PDF) [file pone.0115857.s005.pdf]
